# Identification of Bichalcones as Sirtuin Inhibitors by Virtual Screening and In Vitro Testing

**DOI:** 10.3390/molecules23020416

**Published:** 2018-02-14

**Authors:** Berin Karaman, Zayan Alhalabi, Sören Swyter, Shetonde O. Mihigo, Kerstin Andrae-Marobela, Manfred Jung, Wolfgang Sippl, Fidele Ntie-Kang

**Affiliations:** 1 Department of Pharmaceutical Chemistry, Martin-Luther University of Halle-Wittenberg, Wolfgang-Langenbeck-Str. 4, 06120 Halle (Saale), Germany; karaman.berin@gmail.com (B.K.); zayan82at@hotmail.com (Z.A.); wolfgang.sippl@pharmazie.uni-halle.de (W.S.); 2 Department of Pharmaceutical Chemistry, Faculty of Pharmacy, Biruni University, Istanbul 34010, Turkey; 3 Institute of Pharmaceutical Sciences, Albert-Ludwigs-University of Freiburg, Albertstr. 25, 79104 Freiburg im Breisgau, Germany; soeren.swyter@pharmazie.uni-freiburg.de (S.S.); manfred.jung@pharmazie.uni-freiburg.de (M.J.); 4 Department of Chemistry, University of Kinshasa, Kinshasa P.O. Box 190 XI, Congo; smihigo@yahoo.com; 5 Department of Biological Sciences, Faculty of Science, University of Botswana, Block 235, Private Bag, Gaborone 0022, Botswana; k_marobela@yahoo.com; 6 Department of Chemistry, University of Buea, P.O. Box 63, Buea 00237, Cameroon

**Keywords:** bichalcones, sirtuin inhibitors, virtual screening

## Abstract

Sirtuins are nicotinamide adenine dinucleotide (NAD^+^)-dependent class III histone deacetylases, which have been linked to the pathogenesis of numerous diseases, including HIV, metabolic disorders, neurodegeneration and cancer. Docking of the virtual pan-African natural products library (p-ANAPL), followed by in vitro testing, resulted in the identification of two inhibitors of sirtuin 1, 2 and 3 (sirt1–3). Two bichalcones, known as rhuschalcone IV (**8**) and an analogue of rhuschalcone I (**9**), previously isolated from the medicinal plant *Rhus pyroides*, were shown to be active in the in vitro assay. The rhuschalcone I analogue (**9**) showed the best activity against sirt1, with an IC_50_ value of 40.8 µM. Based on the docking experiments, suggestions for improving the biological activities of the newly identified hit compounds have been provided.

## 1. Introduction

In the last few decades, natural products (NPs) or natural product derivatives have spurred great interest as re-fashionable sources for developing therapeutic agents against human diseases [1,2]. However, the isolation of NPs, the synthesis of their analogues and manufacturing them in larger quantities are major challenges that pharmaceutical companies face today [1,2,3]. Moreover, evaluating the potential of NPs using high throughput screening (HTS) techniques remains problematic [1,4]. Although, combinatorial chemistry is still used to generate large compound libraries for HTS campaigns, a detailed analysis of approved drugs from 1981 to 2014 showed that only two combinatorial chemistry-derived drugs were approved; sorafenib, approved by the Food and Drug Administration (FDA) in 2005 and ataluren, approved by the European Medicines Agency (EMEA) in 2014 [5]. Moreover, it was observed that among all the new therapeutic agents approved during the first 15 years of the 21st Century, about 34% of were either NPs or NP-derived compounds [5].

It had been shown that natural products from African botanical sources possess a unique and broad chemical space, with biological and drug-like properties exploitable in the field of drug discovery [6,7,8,9]. Docking and pharmacophore-based virtual screening (VS) campaigns conducted, for example, against a few selected known anti-cancer drug targets revealed the presence of potential anticancer agents within the newly developed AfroCancer database [6,7]. The efforts of the pan-African Natural Products Library (p-ANAPL) consortium were aimed at collecting physical samples of NPs at a central location, which could then be directly available for screening purposes [10]. Owing to the small quantities of the isolated samples, an efficient approach for the management of the p-ANAPL library has been to only test samples of in silico hits from virtual screening. A previous successful campaign, which combined pharmacophore-based virtual screening and in vitro testing of this compound library, had led to the identification of boldine and ixoratannin as anti-HIV-1 compounds [11]. These findings have prompted the search for potential inhibitors of sirtuins by in silico screening of the p-ANAPL library.

Recently, virtual screening for the identification of sirtuin inhibitors and modulators has received great interest [12,13,14,15,16,17,18,19,20]. Sirtuins are nicotinamide adenine dinucleotide (NAD^+^)-dependent class III histone deacetylases which have been linked to the pathogenesis of numerous diseases like HIV, metabolic disorders, neurodegeneration (including Alzheimer’s disease and Parkinson’s disease) and cancer [21,22,23,24,25,26,27,28]. Since the p-ANAPL library consists of compounds with a broad range of already reported activities, for example, antibacterial, antiviral, anticancer and anti-inflammatory properties [10], this collection represents a good starting point to search for novel inhibitors or modulators of sirtuins. Sirtuins share a highly conserved catalytic core composed of two sub domains; a large NAD^+^ binding domain (Rossmann fold) and a smaller domain generated by two insertions in the NAD^+^ binding domain, together with a helical module and a zinc-binding module [29,30]. 

In the last two decades, several sirt1 and sirt2 crystal structures have been solved in both apo and holo forms [31,32,33,34,35,36,37,38,39,40,41,42,43]. Ternary structures of sirt1 and sirt2 in complex with cofactor analogues, peptide-based and structurally diverse inhibitors revealed a high conformational flexibility in the catalytic pocket, especially in the extended C-pocket region. Thus, the experimentally known structures provide a good coverage of the conformational space of the catalytic pockets of sirt1 and 2 for a target-based vs. campaign. 

In the present work, we used several of the reported crystal structures of sirt1 and sirt2 to virtually screen the p-ANAPL compound collection. The consideration of different protein conformations should increase the chance to identify novel active compounds. Several X-ray structures of sirt1 exist in the Protein Data Bank (PDB), notably co-crystallized with small molecule activators and inhibitors [44]. Sirt2 is also available in complex with a macrocyclic peptide [41], a thiomyristoyl-lysine peptide [39], the cofactors ADP ribose and nicotinamide [40], the inhibitor SirReal2 [40] and the indole derivative EX-243 [38]. These crystal structures were considered for screening. 

## 2. Results

### 2.1. Docking Results

**Figure 1:**
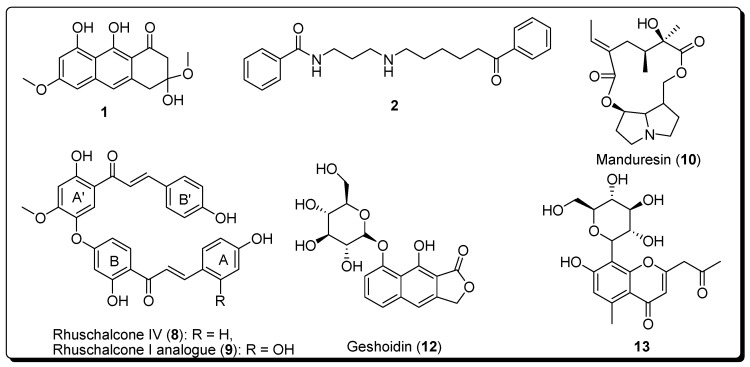
Compounds tested against sirt1, 2 and 3.

Docking of the p-ANAPL virtual compound collection, followed by selecting 5% of the top-ranked poses (using GoldScore) yielded 22 hits for the substrate pocket of sirt1 (PDB ID: 4ZZJ) [45]. From the 22 hits, 5 compounds with sufficient quantities for bioassays were further retained for testing (Figure S1, Supplementary Materials). In a similar fashion, 5% of the top-ranked compounds were selected from docking experiments carried out onto the peptide (PDB IDs: 4R8M and 4L3O), the C (PDB ID: 4RMH) and the extended C (PDB ID: 5D7P) pockets of sirt2. The combined hit lists of sirt1 and sirt2 gave 13 compounds after the removal of duplicates and compounds forming unfavorable conformations within the respective binding sites (Figure S1, Supplementary Materials). Among the 13 retained compounds, only 7 had sufficient amounts within the p-ANAPL collection (>1 mg) for the screening assays. All 7 selected compounds (**1**, **2**, **8**–**10**, **12** and **13**, Figure 1) were tested in the assays against sirt1, 2 and 3. The entire virtual screening process for sirt1 and sirt2 has been summarised in Figure S2 (Supplementary Materials).

### 2.2. In Vitro Activities

Among the selected compounds, **1**, **10**, and **13** showed no inhibitory activities against sirt1, 2, and 3 at 50 µM concentration (Table 1), while inhibition could not be determined for compounds **1** and **12**. Meanwhile, compounds **8** and **9** showed moderate inhibitory effects against both sirt1 (**8**; IC_50_ = 46.7 ± 6.0, **9**; IC_50_ = 40.8 ± 8.5) and sirt2 (**8**; IC_50_ = 48.5 ± 39.5, **9**; IC_50_ = 44.8 ± 5.1) compared to the standard sirtuin inhibitors nicotinamide (NA) and EX-527 (Selisistat). Nevertheless, sirt3 was only slightly affected at a 50 µM concentration by compounds **8** and **9**. Moreover, none of the tested hits showed any PAINS alerts.

## 3. Discussion

**Figure 2:**
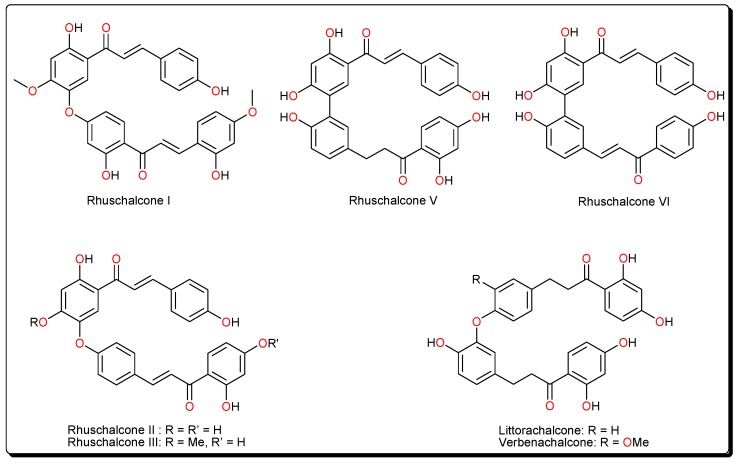
Untested bichalcones from *Rhus* species.

The identified actives are an analogue of rhuschalcone I, along with and rhuschalcone IV. These compounds have been previously isolated from the twigs [46] and root bark [47] of *Rhus pyroides* Burch (Anacardiaceae), respectively. *R. pyroides* is a well known medicinal plant which is widely distributed in Eastern Botswana. This plant is also known to be the sources of several O-linked and C-C coupled bichalcones (Figure 2) and biflavonoids, some of which have been obtained by total synthesis [48,49,50,51,52]. The bichalcones and their analogues are known to possess cytotoxic [47], antiprotozoal [48,49] and carbonic anhydrase inhibitory [50] activities. Meanwhile, biflavones from this plant, e.g., agathisflavone and amentoflavone have shown an affinity for the GABA_A_/benzodiazepine receptor [51]. 

It could be further proposed that analogues of the bichalcones (e.g., the O-linked littorachalcone or verbecharcone, verbenachalcone and rhuschalcones II and III, together with the C-C linked rhuschalcones V and VI, Figure 2) be tested for sirt1, 2 and 3 inhibition. Also, the binding of these compounds in the extended C pocket could be tested in fluorescence assays. It could be suggested that, unlike the rhuschalcones, both C-C and C-O linked non-symmetrical bichalcones be also be synthesized and tested against the sirtuins, with the view of investigating potential selectivities against the isoforms. Besides, chalcones have previously shown deacetylase inhibitory properties against sirt1 and hindered cell growth in HEK293T cells [53].

**Figure 3:**
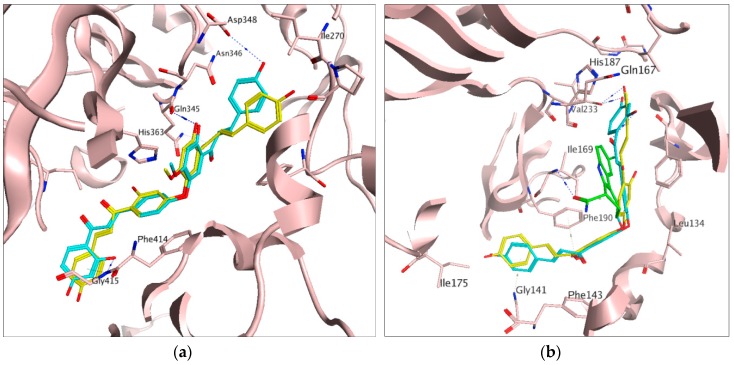
Predicted common binding mode of active compounds in the peptide binding pockets of (**a**) sirt1 (PDB ID: 4ZZJ) and (**b**) sirt2 (PDB ID: 4R8M). In both cases, compound **8** in yellow, compound **9** in cyan, hydrogen bonds drawn as dashed lines, while EX-243 is shown in green on subfigure (**b**).

In order to rationalize the interaction of the identified hits in our study, all docking poses for sirt1 (PDB ID: 4ZZJ) and sirt2 (PDB ID: 4R8M and PDB ID: 5D7P) were analyzed using the Molecular Operating Environment (MOE) program [54]. Docking to sirt1 suggested two possible binding modes for the most active hits, compounds **8** and **9** (Figure 3a and Figure S3). The most favourable (top score) binding mode was observed in the peptide binding pocket, where the hydroxyl group on the ring A of compound **9** interacts with the backbone of the residue Gly415. A similar interaction was also observed for the co-crystallized peptide substrate [45]. Moreover, the hydroxyl groups on the ring A’ of two active compounds made additional H-bonds with the backbone carbonyl group of Gln345 residue. Although compound **8** does not show H-bonding with Asp348, we assume both compounds have the same binding mode, since the experimentally measured inhibitory potencies are very close in all three assays. Moreover, an H-bond interaction was formed between the hydroxyl group of ring B’ of compound **9** and the side chain of the residue Asp348. With regard to binding to the sirt2 peptide pocket, H-bonds were observed between the hydroxyl groups in ring A of the actives and the O atom of Val233 in the protein backbone (Figure 3b). 

The same interactions were observed for the myristol peptide as well in the X-ray structure of Sirt2, but not with the indole derivative EX-243 (Figure 3b). Within the sirt2 extended C pocket (Figure S4), the hydroxyl groups of the B’ ring of the actives interact with His187 via the co-crystallized water molecule HOH676. Meanwhile, the hydroxyl groups of ring A interact with the O atom of Asp 170 in the backbone and the carbonyl groups (near the ring A’) interact with the side chain of IIe232 (Figure S4). Binding in the peptide pockets of both sirt1 and sirt2 is driven by hydrophobic interactions rather than by H-bonding, explaining the similar activities against both sirtuin isoforms.

## 4. Materials and Methods 

### 4.1. Database Preparation

Ligand preparation of the 463 natural compounds in the p-ANAPL database was carried out using the LigPrep module in Schrödinger [55]. 10 low energy conformers were generated for each molecule using the Merck Molecular Forcefield 94 version (MMFF94) [56] implemented in MOE [54] for minimization. Pan-Assay Interference (PAIN) filters were applied using Schrodinger’s Canvas tool [57] and the CbLigand web server [58].

### 4.2. Protein Preparation

All protein X-ray structures were retrieved from the PDB [59]. Protein preparation of the different crystal structures of human sirt1 (PDB IDs: 4I5I [44], and 4ZZJ [45]), was carried out as detailed in the Supplementary Material, while the sirt2 protein structures were prepared as previously described [36] (details in Supplementary Materials). The docking procedure was performed using GOLD program (The Cambridge Crystallographic Data Centre, CCDC, Cambridge, UK) [60,61,62], preceded by preparation of the ligands using the LigPrep (Schrödinger, LLC, New York, NY, USA, 2014) [55] tool in Maestro (Schrödinger, LLC, New York, NY, USA, 2014) [61]. Hydrogen atoms were added to the ligand molecules, followed by minimization, using the MMFFs force field in Maestro [63]. The crystal structure in complex with NAD^+^ (PDB ID: 4I5I), along with the crystal structure co-crystallized with the acetyl lysine peptide (PDB ID: 4ZZJ), were used in the study. The protein structures were protonated and minimized, using the Amber 99SB force field, implemented in MOE [54]. 

### 4.3. Docking, Scoring and Hit Selection

All water molecules, the cofactor and the peptide were removed. The location of the native ligand (NAD^+^ or peptide) was used to define the docking site, where all protein residues within 6 Å from any heavy atom of the respective ligands were considered as part of the binding site. GoldScore was used as the fitness function to score all docking poses. All docking poses were analyzed by visual inspection and some compounds were chosen to be tested by in vitro assays, following a protocol to be given later. In the next step, ligands were docked into the substrate-binding pocket of human sirt1 and sirt2. This was carried out using two different docking programs (Gold [60,61,62] and Maestro [63]). The resulting docking poses were stored. The selection of compounds for testing was carried out by examining protein-ligand interactions in the derived docking poses. In the crystal structures of sirt1 it was shown that substrates make H-bond interactions with the backbone of a conserved valine residue (sirt1 numbering Val412), which is crucial for the correct orientation of the acyl-lysine in the active site [44]. In case of sirt2, the binding interactions of the native ligands including both the peptide substrates, the cofactor fragments and the co-crystallized inhibitors with the protein were first examined [31,32,33,34,35,36,37,38,39,40,41,42,43]. In the hit selection process, special importance was given to compounds interacting with residues Phe234, Phe235, Phe190 and Glu237 in the catalytic pocket. Seven compounds (**1**, **2**, **8**–**10**, **12** and **13**, Figure 1) were identified as hits and retained for testing. All molecules, except the zinc ion (Zn^2+^), were removed from the structures prior to docking. Structural bridging water molecules (where mentioned), were included in the binding site of the protein structures before docking. Docking studies were performed using the Glide program (Schrödinger Suite 2012-5.8) [64,65]. The dockings were done using Glide high-throughput virtual screening (HTVS) mode, treating ligands flexibly. 10 docking poses were calculated for each conformer. Only the top-ranked poses were retained for each compound for each docking run. Docking poses retrieved for the top-ranked 20 compounds (~5% of the whole database) were visually analyzed, the hits being retained based on observed protein-ligand interactions within the target site. In sorting ligand poses by observed protein-ligand interactions, the emphasis was laid on ligand poses with putative interactions within the cofactor (NAD^+^) and peptide binding pockets.

### 4.4. In Vitro Assay

Human sirt1_133–747_ was expressed as a GST-tagged enzyme and purified as described previously [66]. Human sirt2_56–356_ and sirt3_118–395_ were expressed as an N-terminally His_6_-tagged enzyme and purified as described previously. The identity and purity of the produced enzymes were verified using SDS-PAGE [67]. Protein concentration was determined by the Bradford assay [68]. Deacetylase activity of sirtuin isoforms was NAD^+^-dependent and could be inhibited by nicotinamide. Compound samples were provided from the p-ANAPL compound collection in Botswana, which has been stored below 0 °C. The inhibitory activity against hSirt1, hSirt2 and hSirt3 was determined by a histone deacetylase assay, previously established [69], with further details provided in the Supplementary Material. Human sirt1_133–747_, sirt2_25–389_ or human sirt3_118–395_ were mixed with the assay buffer (50 mM Tris, 137 mM NaCl, 2.7 mM KCl, pH 8.0), NAD^+^ (final assay concentration 500 μM), the substrate *Z*-(Ac)Lys-AMC, also termed ZMAL (final assay concentration 10.5 μM).The inhibitor was dissolved in DMSO at various concentrations or DMSO as a control (final DMSO concentration 5% (*v*/*v*)). Total substrate conversion of controls was driven to about 15–30% to assure initial state conditions. The assay was carried out in 96-well plates with a reaction volume of 60 μL per well. All determinations were performed in triplicates. After an incubation for 4 h at 37 °C and 140 rpm, deacetylation was stopped by addition of 60 μL of a solution containing trypsin and nicotinamide (50 mMTris, 100 mMNaCl, 6.7% (*v*/*v*) DMSO, trypsin 16.5 U/μL, 8 mM nicotinamide, pH 8.0). The microplate was further incubated for 20 min at 37 °C and 140 rpm. Finally, fluorescence intensity was measured in a microplate reader (BMG Polarstar, λ_ex_ 390 nm, λ_em_ 460 nm). All compounds were pre-tested on auto-fluorescence, amino-methylcoumarin (AMC) quenching, and trypsin inhibition under assay conditions. Rates of inhibition were calculated by using the controls, containing no inhibitor, as a reference. GraphPad Prism software, version 5 (La Jolla, CA, USA) was employed to determine IC_50_ values. Nicotinamide (as a pan-inhibitor) and EX527 (a sirt inhibitor in clinical trials) were included as controls.

## 5. Conclusions

In the present work, target-based virtual screening was combined with experimental testing in order to identify novel modulators of sirt1 and sirt2 within the p-ANAPL database. Molecular docking studies on available sirt1 and sirt2 crystal structures resulted in two sirt1 and sirt2 inhibitors with moderate inhibitory effect. Although the bichalcones **8** and **9** have been known to possess other biological activities [47,48,49,50,51,52], it is as yet unclear if their cytotoxicities are related to their abilities to inhibit sirtuins. However, natural product libraries like the p-ANAPL and the newly developed NANPDB [70] libraries could be good sources to search for modulators of sirtuins with novel scaffolds.

## Figures and Tables

**Table 1:** IC_50_ or percentage inhibitions at 50% of tested pan-African Natural Products Library (p-ANAPL) compounds against sirt1, 2 and 3.

Compound Number	Sirt 1 (µM)	Sirt 2 (µM)	Sirt 3 (µM or % Inhibition)
1 ^b^	n.d. ^c^	n.d. ^c^	n.d. ^c^
2	n.i. ^a^	n.i. ^a^	n.i. ^a^
8	46.7 ± 6.0	48.5 ± 39.5	38%
9	40.8 ± 8.5	44.8 ± 5.1	23%
10	n.i. ^a^	n.i. ^a^	n.i. ^a^
12 ^b^	n.d.	n.d.	n.d.
13	n.i. ^a^	n.i. ^a^	n.i. ^a^
NA	142.4 ± 9.1	49.8 ± 4.6	67.9 ± 3.3
EX-527	1.4 ± 0.1	10.6 ± 1.1	19%

^a^ n.i. = no inhibition (<10%). ^b^ autofluorescence. ^c^ n.d. = not detectable. Note that activity was not detectable due to the autofluorescence. NA = nicotinamide, EX-527 = sirt inhibitor in clinical trials.

## Supplementary Materials

The following are available online. Figure S1: 13 selected compounds on the hit list: black (>1 mg), red (tested negative) greed (active); Figure S2: Complete workflow of the in silico and in vitro screening processes; Figure S3: Predicted common binding mode of active compounds in the peptide binding pockets of Sirt2 (PDB ID: 4R8M): compound **8** in yellow, compound **9** in cyan, hydrogen bonds drawn as dashed lines; Figure S4: Predicted common binding mode of active compounds in the extended C pocket of Sirt2 (left: PDB ID: 5D7P) and the pocket surface is colored according to hydrophobic (green) and hydrophilic (pink) regions Sirt2 (right). Compound **8** in yellow, compound **9** in cyan, hydrogen bonds drawn as dashed lines; Figure S5: Predicted common binding mode of active compounds in the peptide binding pocket of sirt1 (PDB ID: 4ZZJ), with compound **8** shown in yellow, compound **9** in cyan, hydrogen bonds are drawn as dashed lines and EX-243 in green; Protein Preparation Protocols and detailed in vitro assay description.
