# Supplementary material for: Identification of Bichalcones as Sirtuin Inhibitors by Virtual Screening and In Vitro Testing
Source: Molecules. 2018 Feb 14;23(2):416. doi: 10.3390/molecules23020416 (PMC6017733; doi:10.3390/molecules23020416)
Supplement: Supplementary file 1 [file molecules-23-00416-s001.pdf]

Identification of bischalcones as sirtuin inhibitors by virtual screening and *in vitro* testing

Berin Karaman[1,2,†], Zayan Alhalabi[1,†], Sören Swyter[3], Shetonde O. Mihigo[4], Kerstin Andrae-Marobela[5], Manfred Jung[3], Wolfgang Sippl[1] and Fidele Ntie-Kang,[1,6,*]

[1] *Department of Pharmaceutical Chemistry, Martin-Luther University of Halle-Wittenberg, Wolfgang-Langenbeck-Str. 4, 06120 Halle (Saale), Germany.*
[2] *Department of Pharmaceutical Chemistry, Faculty of Pharmacy, Biruni University, Istanbul, Turkey.*
[3] *Institute of Pharmaceutical Sciences, Albert-Ludwigs-University of Freiburg, Albertstr. 25, 79104 Freiburg im Breisgau, Germany.*
[4] *Department of Chemistry, University of Kinshasa, Kinshasa, DR Congo.*
[5] *Department of Biological Sciences, Faculty of Science, University of Botswana, Block 235, Private Bag, 0022 Gaborone, Botswana.*
[6] *Department of Chemistry, University of Buea, P. O. Box 63, Buea, Cameroon.*
[†] *These authors contributed equally and could be best regarded as joint first authors.*
* *Corresponding author. E-mail address: ntiekfidele@gmail.com (F. Ntie-Kang).*

SUPPLEMENTARY DATA

Figure S1. 13 selected compounds on hit list: black (> 1 mg), red (tested negative) greed (active)

Figure S2. Complete workflow of the *in silico* and *in vitro* screening processes.

Figure S3. Predicted common binding mode of active compounds in the peptide binding pockets of Sirt2 (PDB ID: 4R8M): compound **8** in yellow, compound **9** in cyan, hydrogen bonds drawn as dashed lines).

Figure S4. Predicted common binding mode of active compounds in the extended C pocket of Sirt2 (left, PDB ID: 5D7P) and pocket surface is colored according to hydrophobic (green) and hydrophilic (pink) regions Sirt2 (right). Compound **8** in yellow, compound **9** in cyan, hydrogen bonds drawn as dashed lines).

Figure S5. Predicted common binding mode of active compounds in the peptide binding pocket of sirt1 (PDB ID: 4ZZJ), with compound **8** shown in yellow, compound **9** in cyan, hydrogen bonds drawn as dashed lines and EX-243 in green

Protein Preparation Protocols.

In vitro assay.

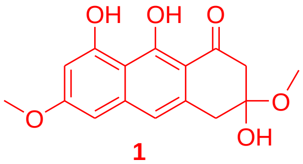

1

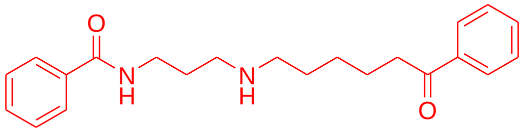

2

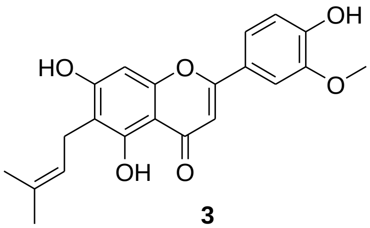

3

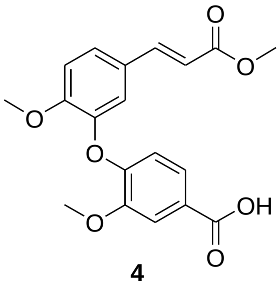

4

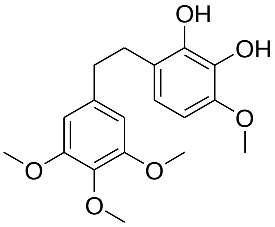

5

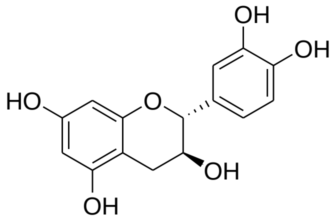

Epicatechin (6)

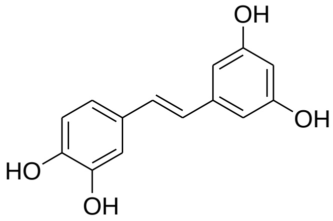

4-(3,5-dihydroxystyryl)benzene-1,2-diol (7)

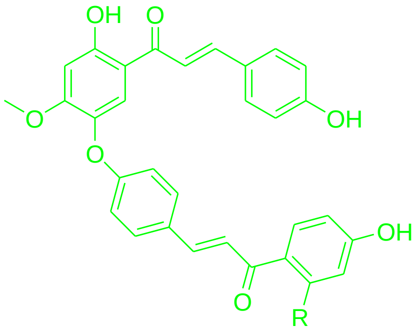

Rhuschalcone IV (8): R = H,
Rhuschalcone I (9): R = OH

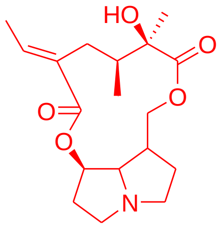

Manduresin (10)

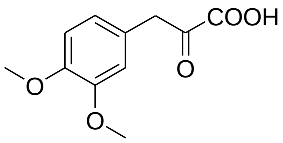

Homoveratric acid (11)

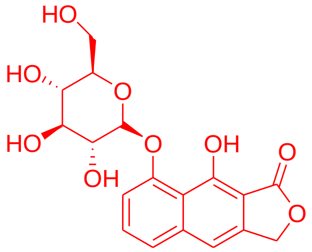

Geshoidin (12)

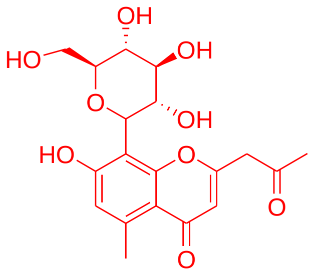

13

Figure S1. 13 selected compounds on hit list: black (> 1 mg), red (tested negative) greed (active)

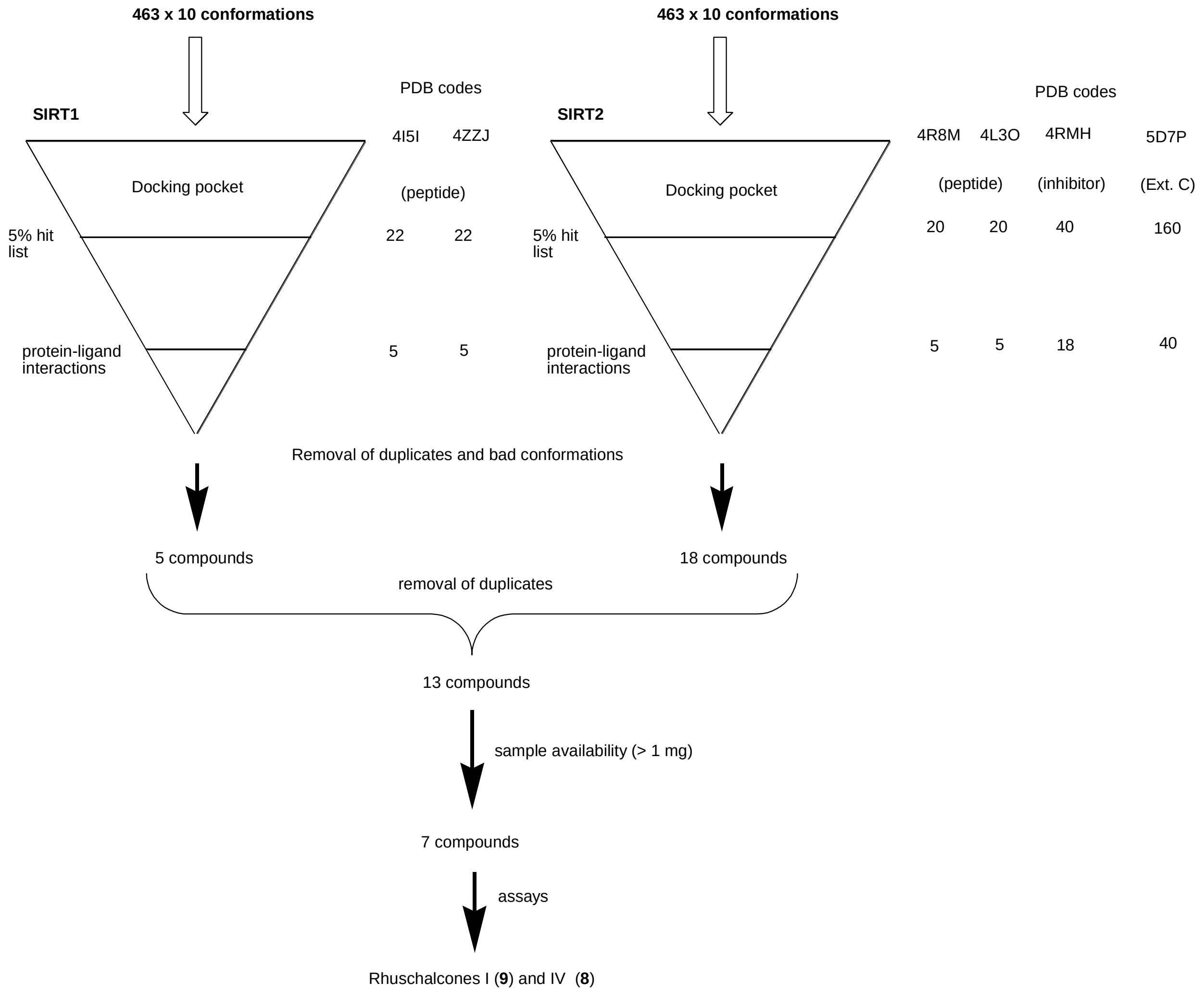

Figure S2. Complete workflow of the *in silico* and *in vitro* screening processes.

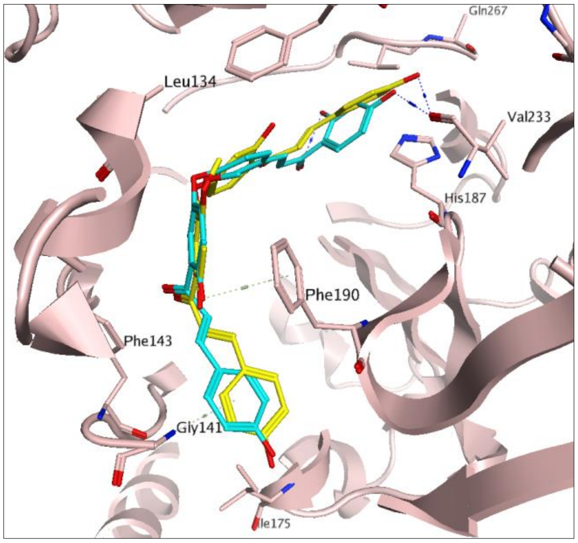

Figure S3. Predicted common binding mode of the active compounds in the peptide binding pockets of Sirt2 (PDB ID: 4R8M): compound **8** in yellow, compound **9** in cyan, hydrogen bonds drawn as dashed lines.

Concerning sirt2, the compound binding modes in the 3 docked pockets were analyzed (Figs. S3 and S4). With regards to binding to the sirt2 peptide pocket, H-bonds were observed between the hydroxyl groups in ring A of the actives and the O atom of Val233 in the protein backbone. The same interactions were observed for the myristol peptide as well in the X-ray structure of Sirt2.[12i] Within the sirt2 extended C pocket (Fig. S4), the hydroxyl groups of the B' ring of the actives interact with His187 *via* the co-crystallized water molecule HOH676. Meanwhile, the hydroxyl groups of ring A interact with the O atom of Asp 170 in the backbone and the carbonyl groups (near the ring A') interact with the side chain of IIe232.

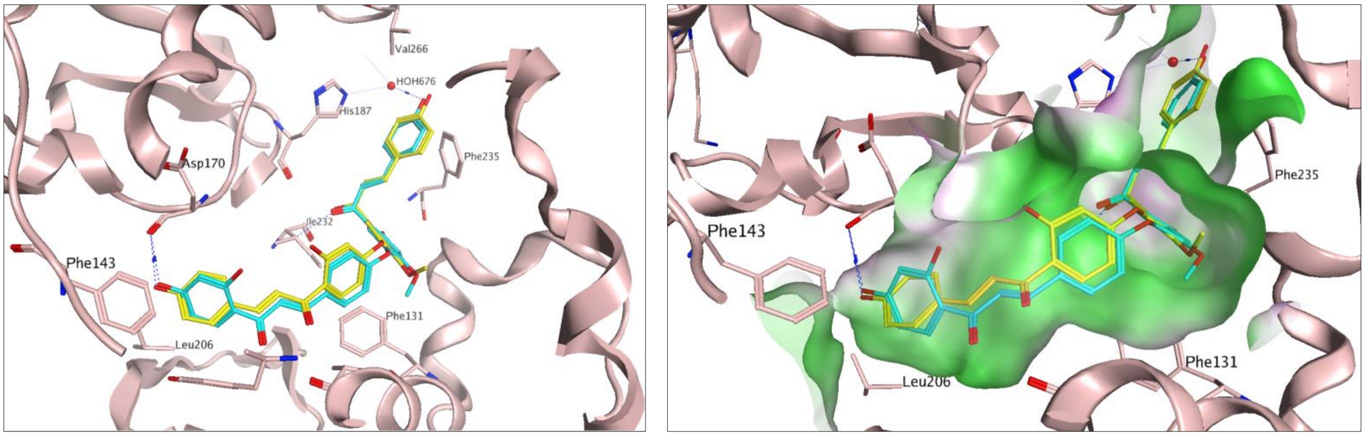

Figure S4. Predicted common binding mode of the active compounds in the extended C pocket of Sirt2 (left, PDB ID: 5D7P) and pocket surface is colored according to hydrophobic (green) and hydrophilic (pink) regions Sirt2 (right). Compound **8** in yellow, compound **9** in cyan, hydrogen bonds drawn as dashed lines.

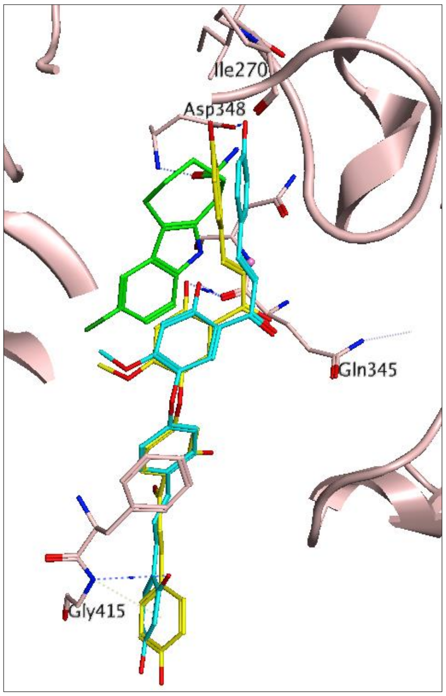

Figure S5. Predicted common binding mode of active compounds in the peptide binding pocket of sirt1 (PDB ID: 4ZZJ), with compound **8** shown in yellow, compound **9** in cyan, hydrogen bonds drawn as dashed lines and EX-243 in green

Protein Preparation Protocols:

Sirt1:

The docking procedure was performed using GOLD program (The Cambridge Crystallographic Data Centre, CCDC, Cambridge, UK) [1-3], preceded by preparation of the ligands using the LigPrep (Schrödinger, LLC, New York, NY, 2014) tool [4] in Maestro (Schrödinger, LLC, New York, NY, 2014) [5]. Hydrogen atoms were added to the ligand molecules, followed by minimization, using MMFFs force field in Maestro [5]. The crystal structure in complex with NAD^+ (PDB ID: 4I5I) and the crystal structure co-crystallized with the acetyl lysine peptide (PDB ID: 4ZZJ) were both taken from the Protein Data Bank (PDB) [6]. The protein structures were protonated and minimized, using the Amber 99SB force field, implemented in MOE [7]. All water molecules, the cofactor and the peptide were removed. The location of the native ligand (NAD^+ or peptide) was used to define the docking site, where all protein residues within 6 Å from any heavy atom of this ligand were considered as part of the binding site. GoldScore was used as the fitness function to score all docking poses. All docking poses were analyzed by visual inspection and some compounds were chosen to be tested by in vitro assays, following a protocol to be given later.

Sirt2:

All molecules, except the zinc ion (Zn^{2+}), were removed from the structures prior to docking. Structural bridging water molecules (where mentioned), were included in the binding site of the protein structures before docking. Docking studies were performed using the Glide program (Schrödinger Suite 2012-5.8) [8-9]. The dockings were done using Glide high-throughput virtual screening (HTVS) mode, treating ligands flexibly. 10 docking poses were calculated for each conformer. Only the top-ranked poses were retained for each compound for each docking run. Docking poses retrieved for the top-ranked 20 compounds (~5% of the whole database) were visually analyzed, the hits being retained based on observed protein-ligand interactions within the target site. In sorting ligand poses by observed protein-ligand interactions, the emphasis was laid on ligand poses with putative interactions within the cofactor (NAD^+) and peptide binding pockets.

In vitro assay:

Human sirt$1_{133\text{-}747}$, sirt$2_{25\text{-}389}$ or human sirt$3_{118\text{-}395}$ were mixed with assay buffer (50 mM Tris, 137 mM NaCl, 2.7 mM KCl, pH 8.0), NAD^+ (final assay concentration 500 μM), the substrate Z-(Ac)Lys-AMC, also termed ZMAL (final assay concentration 10,5 μM), the inhibitor dissolved in DMSO at various concentrations or DMSO as a control (final DMSO concentration 5% (v/v)). Total substrate conversion of controls was driven to about 15% - 30% to assure initial state conditions. The assay was carried out in 96-well plates with a reaction volume of 60 μL per well. All determinations were performed in triplicates. After an incubation for 4 h at 37 °C and 140 rpm, deacetylation was stopped by addition of 60 μL of a solution containing trypsin and nicotinamide (50 mMTris, 100 mMNaCl, 6.7% (v/v) DMSO, trypsin 16.5 U/μL, 8 mM nicotinamide, pH 8.0). The microplate was further incubated for 20 min at 37 °C and 140 rpm. Finally, fluorescence intensity was measured in a microplate reader (BMG Polarstar, λ_{ex} 390nm, λ_{em} 460nm). All compounds were pretested on auto-fluorescence, amino-methylcoumarin (AMC) quenching, and trypsin inhibition under assay conditions. Rates of inhibition were calculated by using the controls, containing no inhibitor, as a reference. GraphPad Prism software (La Jolla, USA) was employed to determine IC_{50} values.

References

1. Jones, G.; Willett, P.; Glen, R. C.; Leach, A. R.; Taylor, R. Development and validation of a genetic algorithm for flexible docking. *J Mol Biol*. **1997**, *267*, 727-748. DOI: 10.1006/jmbi.1996.0897
2. Jones, G.; Willett, P.; Glen, R. C. Molecular recognition of receptor sites using a genetic algorithm with a description of desolvation. *J Mol Biol*. **1995**, *245*, 43-53. DOI: 10.1016/S0022-2836(95)80037-9
3. Verdonk, M. L.; Cole, J. C.; Hartshorn, M. J.; Murray, C. W.; Taylor, R. D. Improved protein-ligand docking using GOLD. *Proteins*. **2003**, *52*, 609-623. DOI: 10.1002/prot.10465
4. LigPrep Software, Version 2.5; LLC: New York, **2011**.
5. Maestro, Version 9.2; LLC: New York, **2011**

6. Berman, H. M.; Westbrook, J.; Feng, Z.; Gilliland, G.; Bhat, T. N.; Weissig, H.; Shindyalov, I. N.; Bourne, P. E. The Protein Data Bank. *Nucleic Acids Res*. **2000**, *28*, 235-42. PMID: 10592235.
7. Molecular Operating Environment, Chemical Computing Group Inc., Montreal, **2014**.
8. Glide program (Schrödinger Suite 2012-5.8)
9. Friesner, R. A.; Banks, J. L.; Murphy, R. B.; Halgren, T. A.; Klicic, J. J.; Mainz, D. T.; Repasky, M. P.; Knoll, E. H.; Shelley, M.; Perry, J. K.; Shaw, D. E.; Francis, P.; Shenkin, P. S. Glide: a new approach for rapid, accurate docking and scoring. 1. Method and assessment of docking accuracy. *J Med Chem*. **2004**, *47*, 1739-1749. DOI: 10.1021/jm0306430
